# Improved allocation strategies for red cell units of blood group O and RhD-negative revealed by current suboptimal utilization

**DOI:** 10.1186/s12967-026-08704-y

**Published:** 2026-08-04

**Authors:** Abdullah Alswied, Willy Albert Flegel

**Affiliations:** Department of Transfusion Medicine, NIH Clinical Center, National Institutes of Health, Bethesda, 20892 MD USA

**Keywords:** Transfusion practices, Inventory management, Blood allocation, Universal donor

## Abstract

**Background:**

Demand for O RhD-negative red cells continues to rise, while overall red cell transfusions have decreased, compounding inventory management challenges. We analyzed O RhD-negative red cell utilization in a large publicly available database.

**Methods:**

Data from 378,855 red cell transfusions were evaluated using the Recipient Epidemiology and Donor Evaluation Study-III (REDS-III). Utilization patterns, recipient demographics, and their correlation with the age of red cell units at transfusion were examined.

**Results:**

The majority of O RhD-negative units were transfused to patients with blood groups other than O RhD-negative (54.5%). A larger percentage of O RhD-negative red cells were transfused after 35 days of collection (41%), compared to other blood groups (18.7%). Patients over 50 years, males, non-O RhD-negative and RhD-positive individuals had higher odds of receiving units stored more than 35 days. Patients with higher hemoglobin concentrations were more likely to receive older blood units.

**Discussion:**

The analysis identified opportunities to improve the allocation of O RhD-negative red cell units, highlighting current inventory practices as suboptimal. The findings call for refined allocation strategies to improve the utilization of this limited resource. The implementation of real-time management approaches may balance the retention of such red cell units with the needs of patients who benefit from the use of RhD-negative blood.

**Supplementary Information:**

The online version contains supplementary material available at https://doi.org/10.1186/s12967-026-08704-y.

## Introduction

Advances in transfusion safety and patient blood management practices have reshaped red cell transfusions [1–3]. Restrictive transfusion thresholds, proactive anemia treatment, improved surgical methods and blood-conserving techniques have lowered the need for red cell transfusions [4–6]. Data from the most recent National Blood Collection and Utilization Survey (NBCUS) show reduced red cell collection and transfusion over the last decade, highlighting the effectiveness of these transfusion safety and patient blood management strategies [7, 8].

While overall red cell utilization has declined, demand for O RhD-negative units continued to rise [9, 10]. The proportion of O RhD-negative units distributed decreased from 11.4% to 9.8% between 2019 and 2021. At the same time, these units accounted for a growing fraction of total red cell transfusions, rising from 11.6% to 12.5%, representing more than 80,000 additional units [10]. In contrast, only 6.9% of US donors are O RhD-negative, leaving blood centers with a limited donor pool to meet an increasing demand [11].

The age of transfused red cell units offers a key signal of inventory practices and reflects how efficiently an organization manages its blood supply [12]. Many European blood suppliers voluntarily opted for 35 days expiration [13], as did the NIH Clinical Center in 2014 [14]. O RhD-negative units often remain in storage longer than units of other blood groups [15, 16]. Organizations can assess turnover and compare these findings with benchmarks by monitoring the storage times. Many studies have described that O RhD-negative units were allocated suboptimally [9, 15–24].

The Association for the Advancement of Blood & Biotherapies (AABB) advises prioritizing O RhD-negative units for RhD-negative patients of childbearing potential [25]. Evidence, however, showed varied adherence to these recommendations [9, 16, 18–20, 22, 23]. Over 40% of O RhD-negative red cell units were transfused to RhD-positive or older patients, who are less likely to require RhD-negative blood [9, 19]. While emergency situations or massive transfusion protocols often justified the use of universal donor units, a significant proportion of O RhD-negative units were allocated for reasons unrelated to clinical necessity, such as preventing outdating or managing inventory [16, 18–20, 22, 23, 26].

Previous studies on red cell utilization have provided important insights despite notable limitations [9, 15–24, 26–28]. Many examined single institutions [20, 27] or specific regions [22, 23], restricting the generalizability of their findings. Several studies had short durations [15–18, 26, 28], missing long-term patterns or seasonal variations. Others relied on aggregated data [17, 19, 24, 28], lacking detailed patient-level information. While some studies investigated aspects of patient demographics [16, 18, 26], shelf life [15, 16, 18, 21, 26], or care settings [9, 16, 19, 20, 26], they did so in isolation or with limited scope. When studies have advanced understanding by integrating multiple factors [9, 16, 18–20, 26], their findings remain constrained by specific contexts and methods.

To overcome limitations of previous studies, we analyzed data from the Recipient Epidemiology and Donor Evaluation Study-III (REDS-III) database [29], which ended in 2016. The database collected laboratory, transfusion, and clinical data from 12 hospitals served by 4 blood centers in the US. Each center acted as a hub, connecting to 2 to 4 institutions, including academic and community hospitals [30]. This dataset offered a multicenter, detailed, and longitudinal perspective for a large, diverse patient population. We concurrently examined the recipients of O RhD-negative red cell units, the age of units at transfusion, and the influence of patient and care settings. Our analysis provides actionable insights and highlights opportunities to improve inventory practices, ensuring these scarce resources benefit the patients who need them most.

## Methods

**Data sources.** We obtained the REDS-III database through the Biologic Specimen and Data Repository Information Coordinating Center [29]. Institutional Review Board approval had been obtained for the database creation, and the data complied with the National Heart, Lung, and Blood Institute data sharing policy. The database for recipients included information on 418,558 transfused red cell units (Fig. S1). ABO incompatible transfusions in 5 cases were identified (0.001% of all analyzed units) and not excluded because their impact on group O blood utilization was negligible. Our study was not considered human subject research (NIH IRB #000576, 20220228).

**Statistical analysis.** Data normality was evaluated with the Shapiro-Wilk test and variances were compared using Levene’s test. Two-sample comparisons were conducted using either the unpaired t-test or Mann-Whitney U test. For multiple comparison groups, one-way analysis of variance (ANOVA) or the Kruskal-Wallis test was used, with post-hoc corrections via the Tukey or Dunnett T3 test, depending on homoscedasticity. Event probabilities between subgroups were compared using the Chi-square test with expected counts derived from marginal totals under the assumption of no association and standardized residuals calculated to quantify deviations. Multivariable logistic regression was conducted to evaluate factors associated with the transfusion of red cell units older than 35 days. Variables included in the model were recipient age, sex, blood group, RhD status, and care setting. Odds ratios (OR) with 95% confidence intervals (CI) were calculated for each variable.

Statistical analysis was performed using Prism 9 (GraphPad Software 9.5.0, Carlsbad, CA) and Python version 3.10.0 (Python Software Foundation, Wilmington, DE). For extensive data processing and computationally intensive tasks, the NIH Biowulf high-performance computing cluster was employed. Statistical significance was defined as *p* < 0.05 and indicated by asterisks: *p < 0.05, **p < 0.01, ***p < 0.001, ****p < 0.0001. Non-significant outcomes were labeled ‘ns’.

## Results

The publicly available REDS-III recipient database contained records of 418,558 red cell transfusions across all blood groups from 2013 to 2016. After excluding incomplete entries and patients under 15 years of age, we analyzed 378,855 transfusions (Fig. S1).

**Donor demographics.** The frequency of red cell donation varied by blood type, with O RhD-negative donors having the highest rate at 0.80 donations per year. Only 8.2% of all donors were O RhD-negative (Table 1).

**Table 1:** Distribution of blood groups and donation frequency among blood donors of red cell units

Blood group	Donations per year (mean) *	Donors	
		n	%
O RhD-negative	0.80	46,027	8.2%
B RhD-negative	0.74	10,218	1.8%
A RhD-negative	0.73	32,526	5.8%
AB RhD-negative	0.70	3,815	0.7%
A RhD-positive	0.68	173,802	31.2%
O RhD-positive	0.66	210,667	37.8%
B RhD-positive	0.65	60,404	10.8%
AB RhD-positive	0.62	20,573	3.7%

^*^Annual donation frequency, mean over 4-year study period

**Fig. 1:**
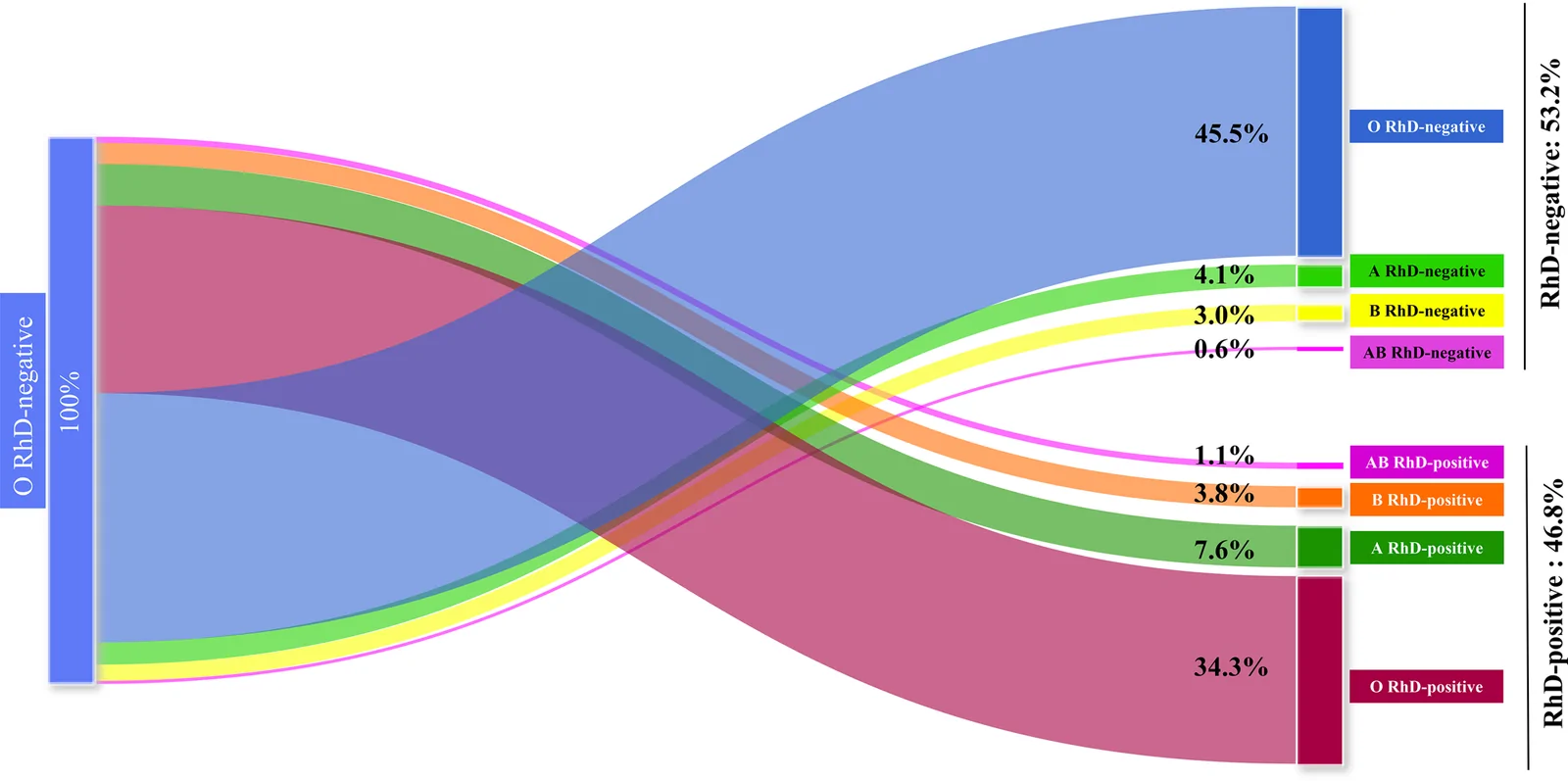
Sankey diagram illustrating the allocation of O RhD-negative red cell units to patients by ABO and RhD blood group. The width of each band indicates the relative proportion of units transfused to each group. A total of 45.5% of the units were transfused to O RhD-negative patients and 34.3% of them to O RhD-positive patients

**Overall red cell utilization trends and patient blood groups.** The overall utilization declined by 13.9% during the 4-year of the study, while the proportion of O RhD-negative units transfused increased (Fig. S2). The majority of O RhD-negative units (54.5%) were transfused to patients with other blood groups, 46.8% of these units to RhD-positive patients (Fig. 1).

**Age of red cell units at transfusion.** O RhD-negative units were transfused at a median age of 33 days, significantly older than any RhD-positive units (Table 2), whether blood group AB, B, A or O. A higher percentage of O RhD-negative units (41%) were transfused after 35 days of collection, compared to 18.7% (mean, range 12.7%– 42.2%) across all ABO groups.

**Table 2:** Age of red cell unit at transfusion

Blood group	Age at transfusion (days)			Units older than 35 days	
	Median	IQR	P *	%	P*
AB RhD-negative	33	22–39	<0.0001	42.2%	<0.0001
B RhD-negative	33	22–38	<0.0001	40.6%	ns
O RhD-negative	33	19–38	Reference	41.0%	Reference
A RhD-negative	32	20–38	ns	37.5%	ns
AB RhD-positive	32	22–38	<0.0001	36.8%	<0.0001
B RhD-positive	25	15–35	<0.0001	22.1%	<0.0001
A RhD-positive	24	15–33	<0.0001	17.3%	<0.0001
O RhD-positive	21	13–31	<0.0001	12.7%	<0.0001

^*^all blood groups compared to O RhD-negative (reference)

IQR – interquartile range

ns – not significant

**Fig. 2:**
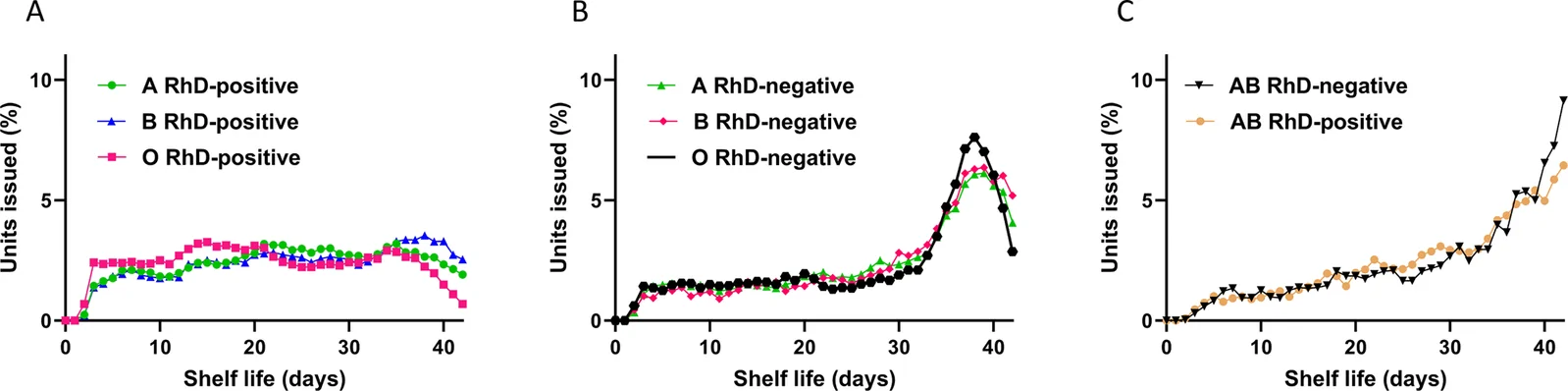
Red cell unit transfusion by the age of the units with a maximum shelf-life of 42 days. (**A**) A RhD-positive, B RhD-positive, and O RhD-positive units maintain a consistent issuance rate, with a slight decline for O RhD-positive beyond day 35 after donation. (**B**) A RhD-negative, B RhD-negative and O RhD-negative units had a stable rate for the first 30 days, then peaked at 37–39 days after donation. (**C**) AB red cell units had a comparable issuance rate independent of RhD type with a marked rise toward the end of their shelf-life

There were differences in the transfusion pattern depending on blood group during the 42-day shelf life (Fig. 2). Units with any of the 3 prevalent ABO types had a stable issuance rate during their shelf life if they were RhD-positive (Fig. 2A). By contrast, a sharp rise was observed after 30 days of shelf life for units of these blood groups when they were RhD-negative (Fig. 2B). Only units of blood group AB showed a comparable rise at the end of shelf life, independent of the RhD type (Fig. 2C).

**Fig. 3:**
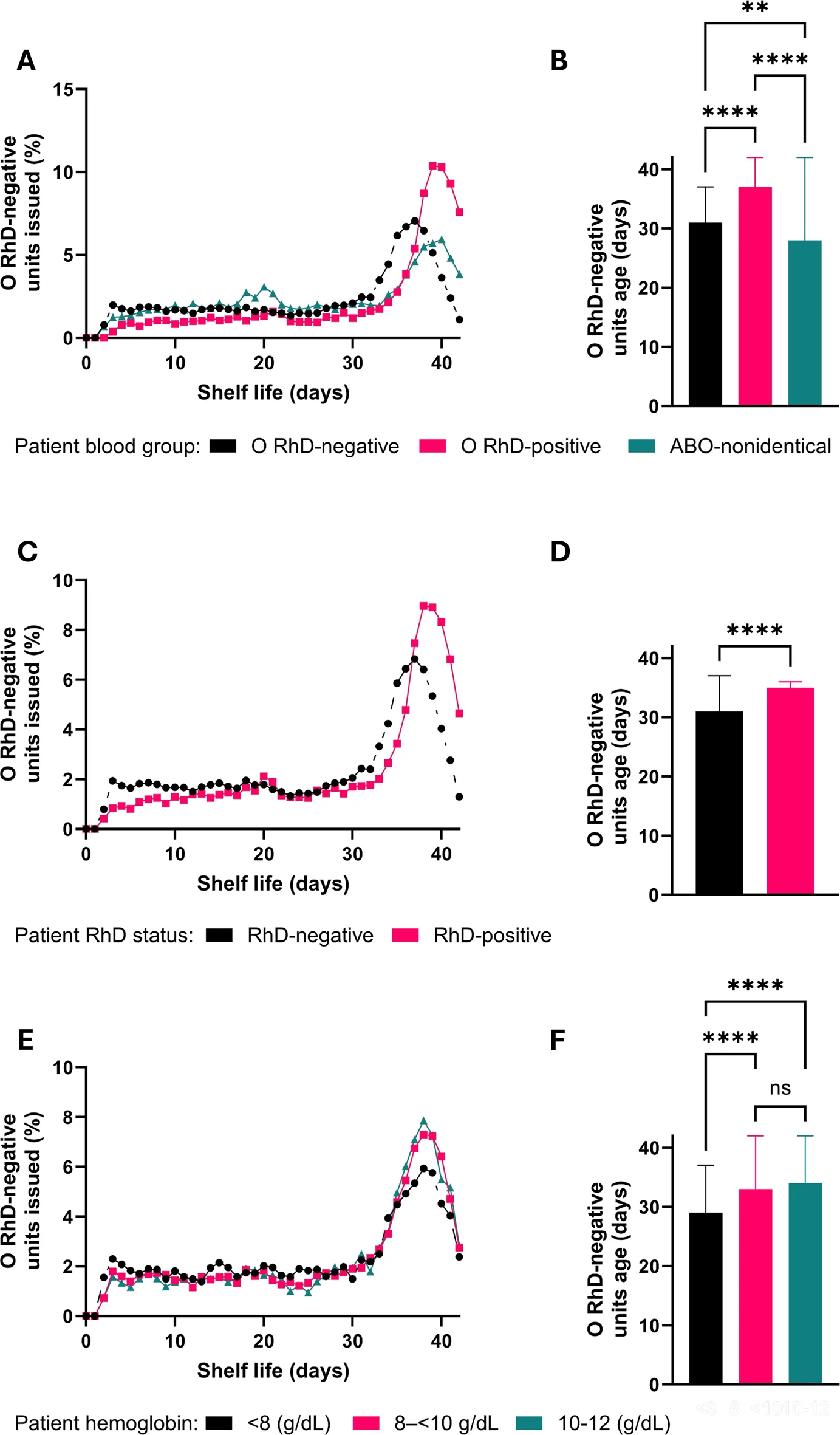
Patient factors influencing the age of O RhD-negative units at transfusion. (**A**) Distribution of units transfused over time after donation for different patient groups. Peaks occurred at 37 days for O RhD-negative, 39 days for O RhD-positive, and 40 days for ABO RhD-nonidentical patients. (**B**) Median age of red cell units by patient group. O RhD-negative patients received units with a median age of 31 days (IQR 16–37). In contrast, O RhD-positive patients received significantly older units (median 37 days, IQR 26–39; *p* < 0.0001), whereas ABO RhD-nonidentical patients were given units with a median age of 28 days (IQR 17–38; *p* < 0.0014). (**C**) The distribution of units transfused post-donation peaked at Day 37 for RhD-negative patients and Days 38–39 for RhD-positive patients. (**D**) Age of transfused red cell units by patient RhD status. RhD-negative patients received units with a median age of 31 days, (IQR 16–37), whereas RhD-positive patients had significantly older units (median 35 days, IQR 21–39; *p* < 0.0001). (**E**) Age of transfused red cell units stratified by patient hemoglobin concentration. All groups shared similar patterns, with peaks around 37–39 days after donation. (**F**) Median age of transfused units stratified by hemoglobin concentration. The 10–12 g/dL group received the oldest units (median 34 days, IQR 19–38). Next was the 8–<10 g/dL group (median 33 days, IQR 18–38; *p* = 0.1828 vs. 10–12 g/dL). The < 8 g/dL group received the units with a median age of 29 days (IQR 15–37), which were significantly lower than those in the other two groups (*p* < 0.0001)

**Fig. 4:**
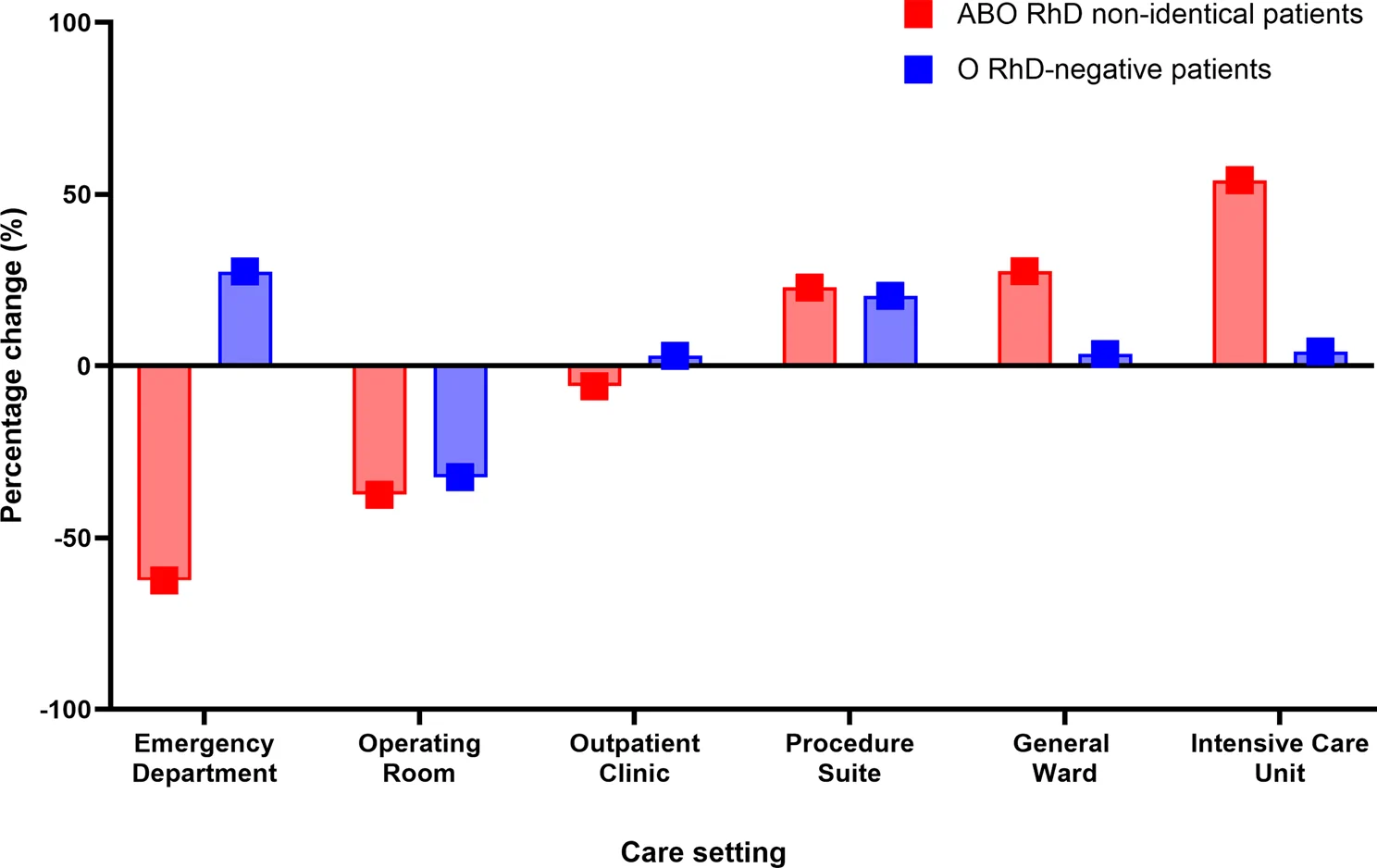
Allocation of O RhD-negative units in different patient care settings. Percentage change in the use of O RhD-negative units older than 35 days compared with those stored 35 days or shorter, across 6 different care settings. Negative percentages indicate decreased use of units stored more than 35 days in that setting; positive percentages indicate an increase. The data is stratified by patient blood groups: ABO/RhD-nonidentical (red bars) versus O RhD-negative (blue bars)

**Factors influencing the age of O RhD-negative units at transfusion. (i) Blood groups.** The units’ age correlated with the patients’ ABO blood group and RhD antigen (Fig. 3). The age of O RhD-negative units at transfusion peaked between 37 and 40 days, depending on the patients’ blood group (Fig. 3A). The units transfused to O RhD-positive patients were significantly older than those transfused to O RhD-negative patients (Fig. 3B). The transfusion rate rose sharply for O RhD-negative units beyond day 30 (Fig. 3C); RhD-negative patients received them significantly earlier (*p* < 0.0001) than the RhD-positive patients (Fig. 3D). **(ii) Hemoglobin.** The units’ age correlated with the patients’ hemoglobin concentration (Hb) before transfusion (Fig. 3E). Patients with Hb below 8 g/dL received units at 29 days (median, IQR 15–37) and those with Hb between 10 and 12 g/dL at 34 days (median, IQR 19–38), a significant difference (*p* < 0.0001) in the large patient cohort (Fig. 3F). **(iii) Patient care setting.** The O RhD-negative units’ age correlated with the care setting where the patient was transfused (Fig. 4). For patients other than O RhD-negative, units stored 35 days or longer were transfused 62.4% less frequently in the Emergency Department compared to units stored 35 days or shorter. Such units older than 35 days were transfused more frequently in General Wards and Intensive Care Unit (27.6% and 54.1%, respectively). These patterns almost reversed when O RhD-negative patients were transfused.

**Predictors of red cell units transfused past 35 days.** Multivariable logistic regression indicated (Table 3) that male sex, age over 50 years, and having either a non-O RhD-negative blood group or RhD-positive status were independently linked to higher odds of receiving units older than 35 days. These findings were also seen when we stratified the O RhD-negative units by sex, age, and RhD status (Fig. S3).

**Table 3:** Factors influencing transfusion of red cell units older than 35 days: multivariable logistic regression

Variable	Odds ratio (95% CI)	P
Age		
≤50 years	–	–
>50 years	1.72 (1.64–1.80)	<0.0001
Sex		
Female	–	–
Male	1.05 (1.01–1.09)	0.0168
ABO group		
Non-O RhD-negative	–	–
O RhD-negative	0.78 (0.72–0.85)	<0.0001
RhD status		
RhD-positive	–	–
RhD-negative	0.51 (0.47–0.54)	<0.0001
Care setting		
Emergency Department	0.43 (0.39–0.48)	<0.0001
Intensive Care Unit	1.18 (1.06–1.32)	0.0022
Operating Room	0.63 (0.56–0.72)	<0.0001
General Ward	1.08 (0.97–1.20)	ns
Outpatient Clinic	0.88 (0.78–0.99)	0.0408
Procedure Suite	1.03 (0.83–1.27)	ns

CI – Confidence Interval

ns – not significant

**O RhD-negative patients receiving O RhD-positive red cell units.** The 3,039 O RhD-positive units transfused to O RhD-negative patients represented 0.8% of all units in this study (Table 4). Most (82.5%), but not nearly all, were transfused to patients older than 50 years, with a male preponderance even in this age cohort. Comparisons across demographic groups showed a significant association between patient demographics and the likelihood of receiving O RhD-positive units (χ^2^ (3) = 517.2, *p* < 0.0001). Sex was the primary driver of this difference (χ^2^ (1) = 459, *p* < 0.0001), while age had a smaller but still significant effect (χ^2^ (1) = 24, *p* < 0.0001). Standardized residuals (Table S1) indicated that males, particularly those over 50, received O RhD-positive red cell units more frequently than expected, while females in both age groups received them at lower-than-expected rates.

**Table 4:** O RhD-negative patients receiving RhD-positive red cell units

		Units transfused (n)								
Patient demographics		Per patient					Recipients		Encounters	
		Median	IQR	Range	Total	%	n	%	n	%
Male										
>50 years old		2	1 - 4	1 - 49	1548	50.9%	436	49.4%	544	50.3%
≤50 years old		2	2 - 4	1 - 61	449	14.8%	88	9.9%	110	10.2%
Female										
>50 years old		2	1 - 3	1 - 22	960	31.6%	344	39%	398	36.8%
≤50 years old		3	2 - 4.5	1 - 32	82	2.7%	15	1.7%	29	2.7%
Total					3,039	100%	882	100%	1081	100%

## Discussion

O RhD-negative individuals donated blood more frequently than any other donors. However, more than half of their donated red cell units were transfused to patients with blood groups other than O RhD-negative, and nearly half of these patients were RhD-positive. O RhD-negative units, in difference to any other blood type, were more often transfused after 35 days of storage. The sharp rise in O RhD-negative usage after 35 days was linked to demographic factors, such as older age and male sex. The older the unit’s age at transfusion the higher were the patients’ hemoglobin before transfusion. All these observations point to decision processes that are actionable and can be optimized.

We analyzed red cell utilization at both a multi-center and patient-level scale using the large, publicly available, and systematically collected REDS-III database. This national-level resource covered multiple institutions, diverse populations, and several years. Since REDS-III is available and rigorously standardized, other researchers can replicate and extend our findings, apply different analytical approaches, or investigate additional questions reflecting the database’s broad potential to drive ongoing advancements in transfusion medicine.

More than half of O RhD-negative units (54.5%) were transfused to patients who were not O RhD-negative, including 46.8% who were RhD-positive (Fig. 1). This finding aligned with earlier work indicating that 28% to 67% of O RhD-negative units are transfused to non-O RhD-negative patients [16, 17, 31]. Many of these units were transfused to male patients, and those older than 50 years, or RhD-positive (Fig. S3), also consistent with earlier research [16, 18, 21, 23, 25]. While some of these patients may have needed O RhD-negative units, many did not require them.

A relevant number of 15 O RhD-negative women of childbearing age (1.7%) received RhD-positive units. Each transfusion posed a considerable risk of alloimmunization (Table 4), often exceeding 50% [32–36]. Men, especially those older than 50 years, received O RhD-positive units far more frequently than expected (Table S1), compared to women of the same age group. This observation documented a deviation from recommendations, implying an overly cautious stance toward female patients, not based on medical need [2, 25]. Persistent challenges in balancing inventory logistics with patient safety are exemplified by this inconsistent provision of O RhD-negative units to females of childbearing potential coupled with overutilization in patients who may not require them.

Our report, in line with several other studies on REDS-III, could not extend beyond 2016, when REDS-III ended. Little evidence published during the 10 years since pointed to relevant changes in the practice [37] that has been analyzed in our current study. However, studying more recent periods, other regions and different transfusion practices may well be warranted.

O RhD-negative units were transfused at an older unit’s age than other blood groups, except for AB and B negative (Table 2). Of all units, 81.3% were used by day 35, a similar to 80.9% reported by NBCUS [7, 8]. However, only 59% of O RhD-negative units were used by day 35, indicating differential inventory management practices [10]. Patients with higher hemoglobin concentrations received older units (Figs. 3E and F) [18, 19]. Patients aged 50 years and older, males, non-O RhD-negative and RhD-positive individuals had higher odds of receiving near-expiry units (Table 3). These units were primarily used in care settings where the patient’s ABO and RhD are typically known in advance (Fig. 4) and not in the Emergency Department. The shift in usage of near-expiry units from emergency settings to General Wards and Intensive Care Unit indicated a deliberate allocation strategy. This pattern suggested that near-expiry O RhD-negative units were being allocated to less urgent clinical scenarios and to patients with known blood types [16, 18, 19]

Actionable strategies have emerged from our analysis: (i) Prioritizing O RhD-negative units for RhD-negative individuals, especially females of childbearing potential or those with unknown RhD status, preserves this universal donor resource for those who need it most. (ii) Enforcing stricter first-in, first-out policies, complemented by real-time inventory monitoring and dynamic expiration thresholds, mitigates sudden surges in usage as units approach expiration. (iii) Tailoring transfusions to clinical urgency by minimizing near-expiry units for non-urgent cases reduces unnecessary transfusions. (iv) Adhering to established protocols such as maximum surgical blood orders, limiting on-hold times, and redistributing soon-to-expire stock among networked facilities helps maintain balanced inventories. (v) Finally, advanced data analytics and predictive modeling that incorporate patient demographics, care setting, and hemoglobin triggers can forecast demand and guide timely inventory replenishment.

Key trends were identified; however, the findings of our analysis may not fully capture recent transfusion practices or updated recommendations. Transfusion medicine is rapidly evolving, and recent initiatives may have influenced O RhD-negative red cell utilization since the REDS-III data were collected. Nonetheless, our findings remain relevant, given ongoing challenges in meeting O RhD-negative demand, as documented by the AABB’s weekly blood supply reports [38].

Although O RhD-negative donors accounted for only 8.2% of the donor population, they had the highest donation frequency at 0.8 donations per donor (Table 1), reflecting both strong demand and reliance on a relatively small donor base. Despite targeted strategies, the growing demand and limited supply of O RhD-negative blood suggest that current efforts may be insufficient, intensifying the need for judicious use of these units to ensure they reach the patients who need them most [16, 18, 39, 40].

## Electronic supplementary material

Below is the link to the electronic supplementary material.

Supplementary material 1

Supplementary material 2

## Data Availability

All underlying data are part of the REDS-III database, which is available through the Biologic Specimen and Data Repository Information Coordinating Center.

## Declarations

### Disclaimer

This research was supported in part by the Intramural Research Program of the National Institutes of Health (NIH). The contributions of the NIH authors are considered Works of the United States Government. The findings and conclusions presented in this paper are those of the authors and do not necessarily reflect the views of the NIH or the U.S. Department of Health and Human Services.
