# Supplementary material for: Improved allocation strategies for red cell units of blood group O and RhD-negative revealed by current suboptimal utilization
Source: J Transl Med. 2026 Aug 4;24:1276. doi: 10.1186/s12967-026-08704-y (PMC13653384; doi:10.1186/s12967-026-08704-y)
Supplement: Supplementary file 1 [file 12967_2026_8704_MOESM1_ESM.pdf]

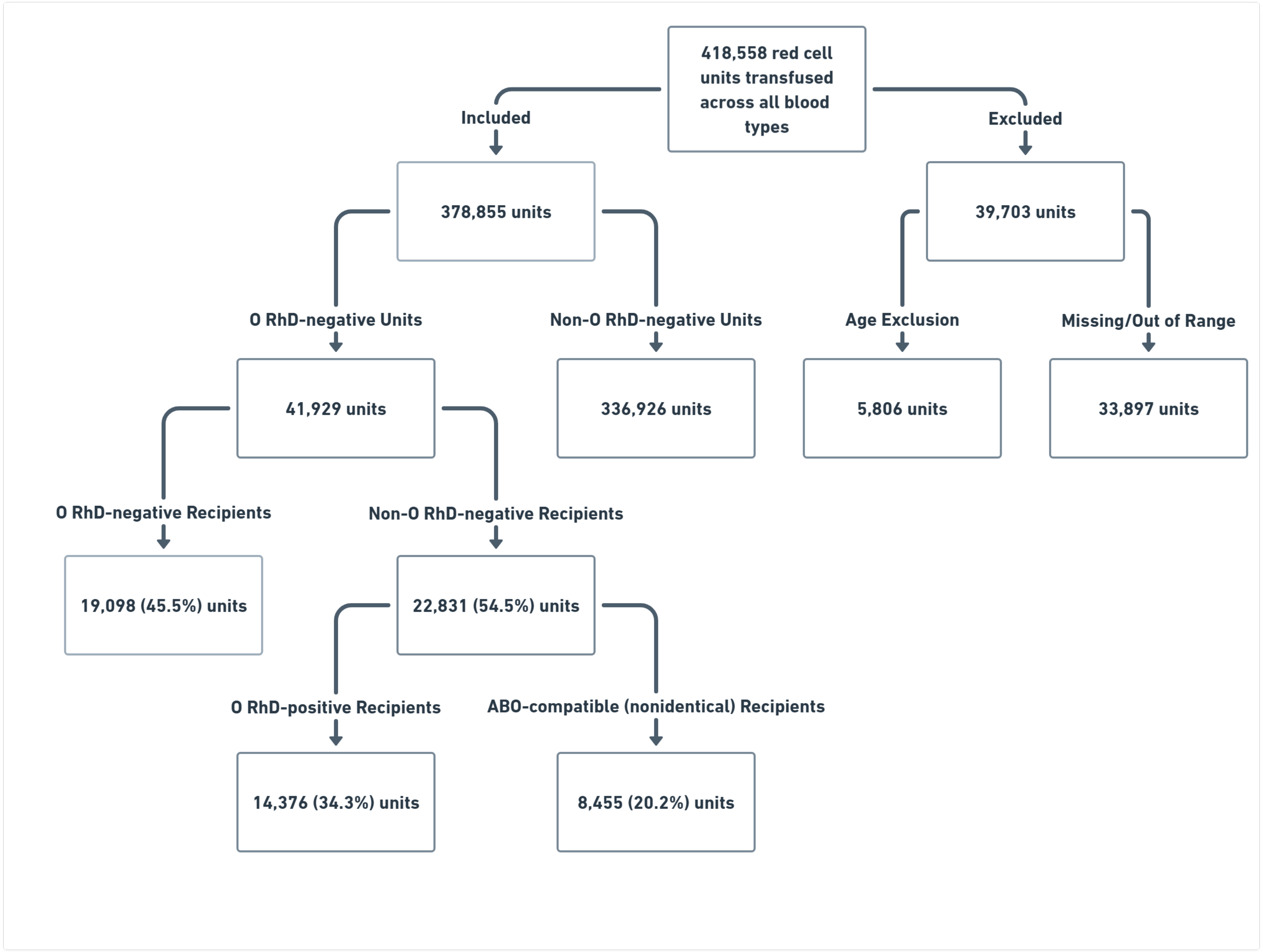
418,558 red cell units transfused across all blood types
Included
Excluded
378,855 units
39,703 units
O RhD-negative Units
Non-O RhD-negative Units
Age Exclusion
Missing/Out of Range
41,929 units
336,926 units
5,806 units
33,897 units
O RhD-negative Recipients
Non-O RhD-negative Recipients
19,098 (45.5%) units
22,831 (54.5%) units
O RhD-positive Recipients
ABO-compatible (nonidentical) Recipients
14,376 (34.3%) units
8,455 (20.2%) units

Figure S1

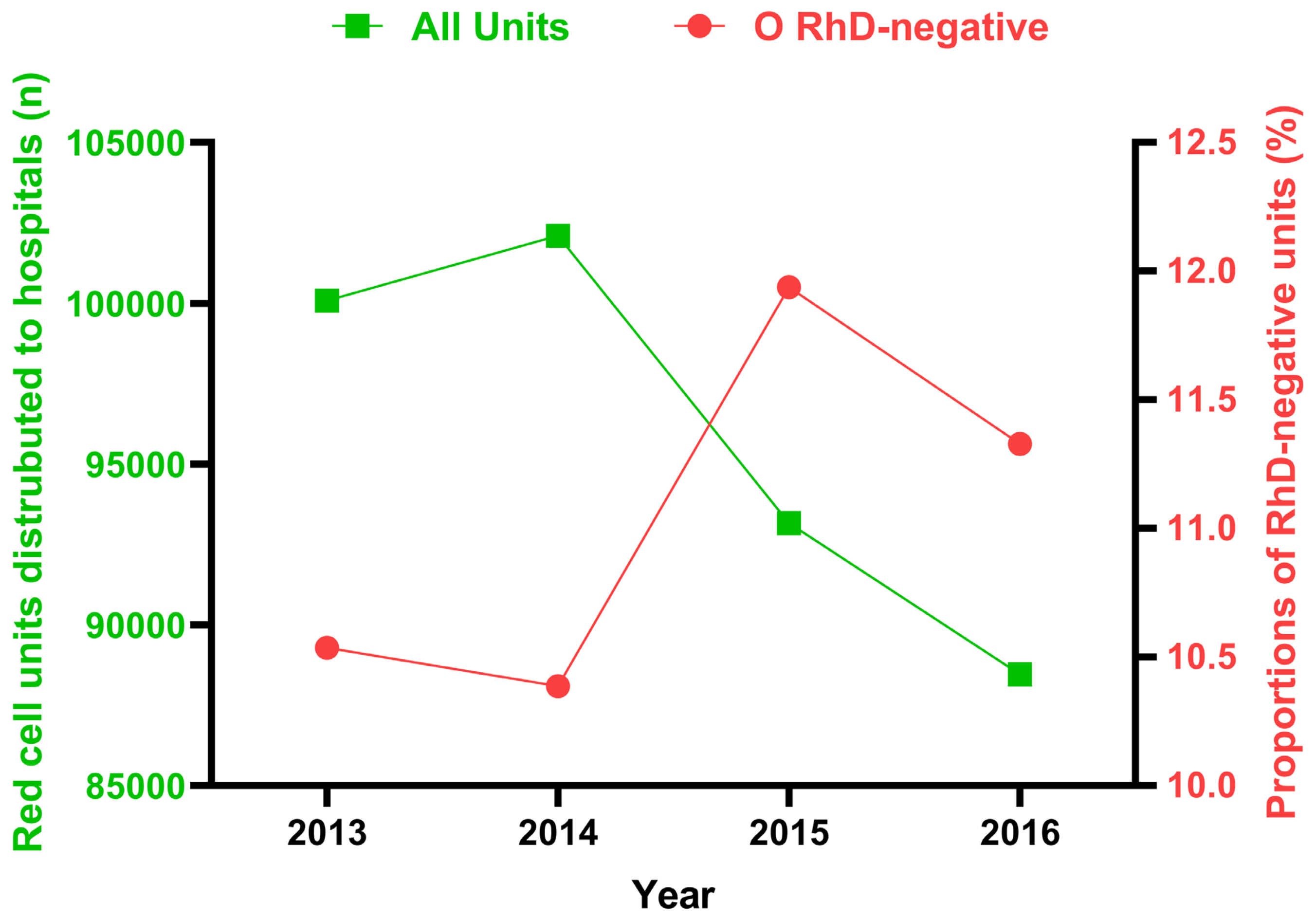
All Units
O RhD-negative
Red cell units distrubuted to hospitals (n)
105000
100000
95000
90000
85000
Proportions of RhD-negative units (%)
12.5
12.0
11.5
11.0
10.5
10.0
2013
2014
2015
2016
Year

Figure S2

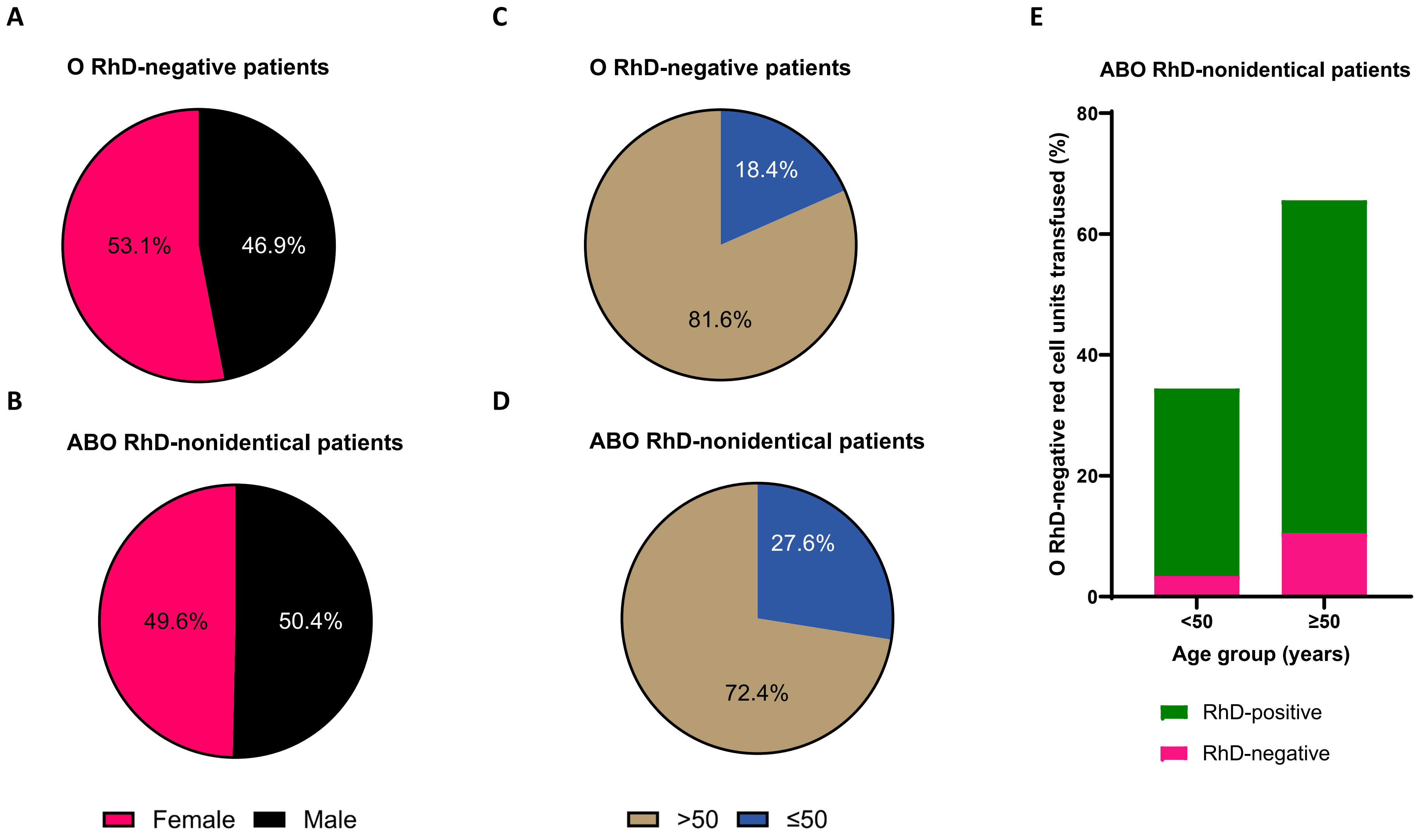
A
O RhD-negative patients
53.1%
46.9%
B
ABO RhD-nonidentical patients
49.6%
50.4%
Female
Male
C
O RhD-negative patients
18.4%
81.6%
D
ABO RhD-nonidentical patients
27.6%
72.4%
>50
≤50
E
ABO RhD-nonidentical patients
O RhD-negative red cell units transfused (%)
80
60
40
20
0
<50
≥50
Age group (years)
RhD-positive
RhD-negative

Figure S3
