# Supplementary material for: Improved allocation strategies for red cell units of blood group O and RhD-negative revealed by current suboptimal utilization
Source: J Transl Med. 2026 Aug 4;24:1276. doi: 10.1186/s12967-026-08704-y (PMC13653384; doi:10.1186/s12967-026-08704-y)
Supplement: Supplementary file 2 [file 12967_2026_8704_MOESM2_ESM.pdf]

Table S1. Observed versus expected transfusions of O RhD-negative patients receiving RhD-positive red cell units

Patient demographics	O RhD-positive		
	Observed	Expected	Standard Residual
Male			
>50 years old	1548	1108	+13.22
≤50 years old	449	341.3	+5.79
Female			
>50 years old	960	1297	-9.36
≤50 years old	82	291	-12.25

Supplementary Material

Figure S1. Study inclusion and exclusion work flowchart. A total of 418,558 units were transfused in the REDS-III database across all blood groups. After excluding patients younger than 15 years and missing or out-of-range parameters, 378,855 units remained for analysis (see Fig. 1 and Results).

Figure S2. Utilization of O RhD-negative red cell units between 2013 and 2016. The proportion of O RhD-negative units (red) varied over the study period and was 11.5% in 2013 (11.4% in 2014, 13.1% in 2015, and 12.5% in 2016). The total number of O RhD-negative units distributed declined from 99,446 in 2013 to 85,531 in 2016 (green).

Figure S3. Transfusion of O RhD-negative red cell units to RhD-negative and ABO RhD-nonidentical patients, stratified by sex (A and B), age (C and D), and RhD type (E). The distribution differs between patients of O RhD-negative (A & C) and any other blood group (B and D). RhD-positive patients constituting the majority of recipients in the ABO RhD-nonidentical cohort (E).

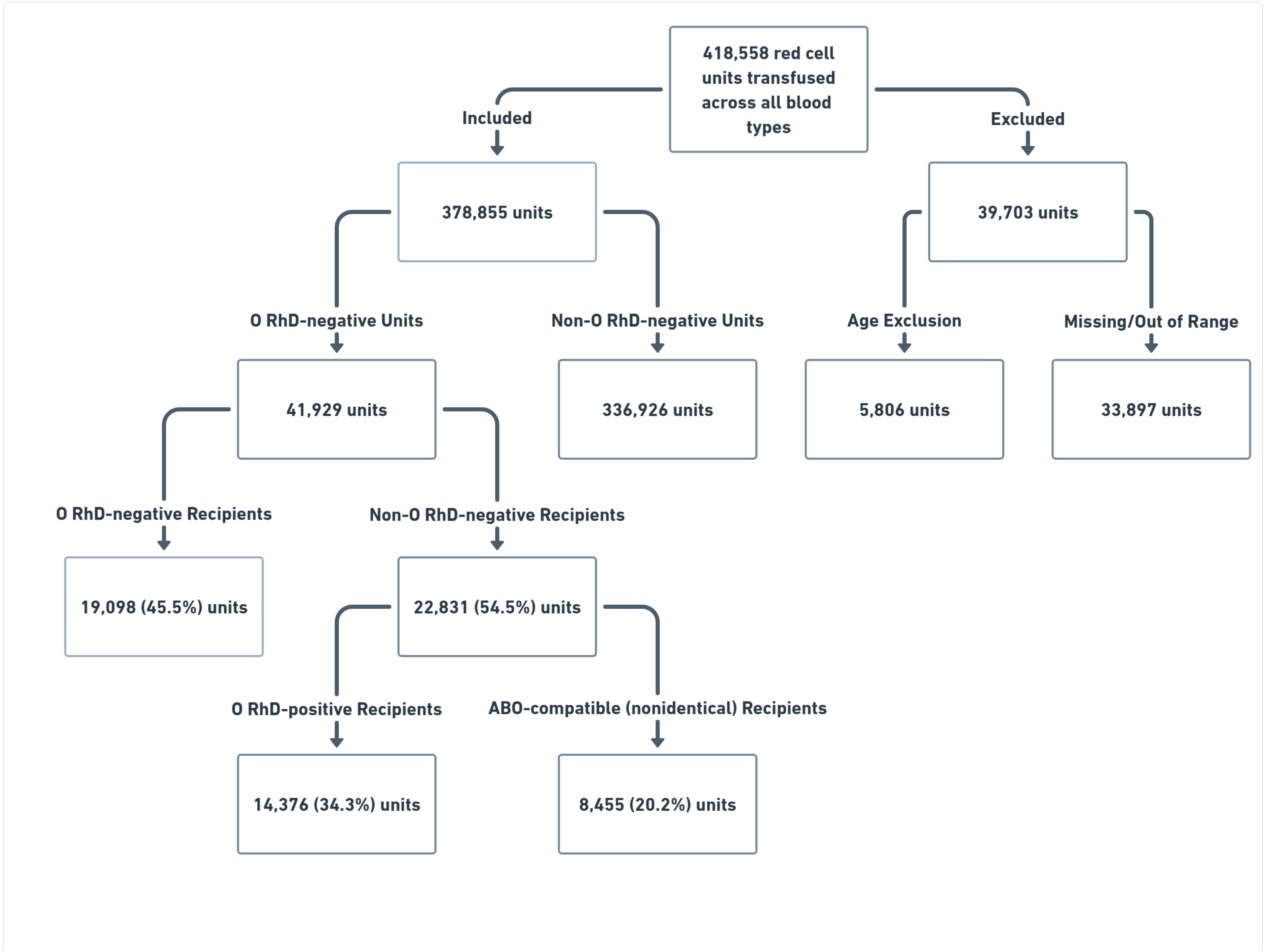
418,558 red cell units transfused across all blood types
Included
Excluded
378,855 units
39,703 units
O RhD-negative Units
Non-O RhD-negative Units
Age Exclusion
Missing/Out of Range
41,929 units
336,926 units
5,806 units
33,897 units
O RhD-negative Recipients
Non-O RhD-negative Recipients
19,098 (45.5%) units
22,831 (54.5%) units
O RhD-positive Recipients
ABO-compatible (nonidentical) Recipients
14,376 (34.3%) units
8,455 (20.2%) units

Figure S1

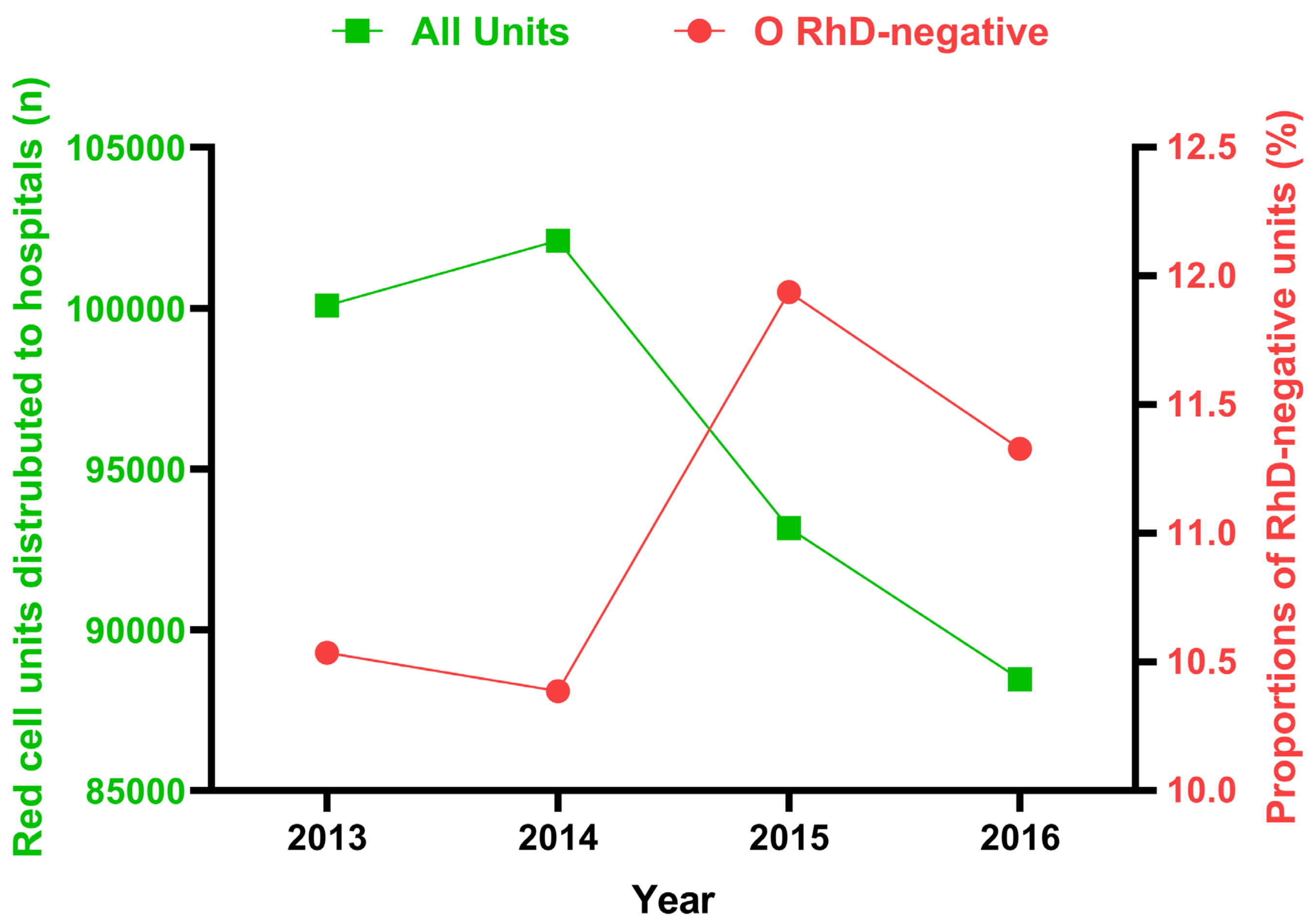
All Units
O RhD-negative
Red cell units distrubuted to hospitals (n)
105000
100000
95000
90000
85000
12.5
12.0
11.5
11.0
10.5
10.0
Proportions of RhD-negative units (%)
2013
2014
2015
2016
Year

Figure S2

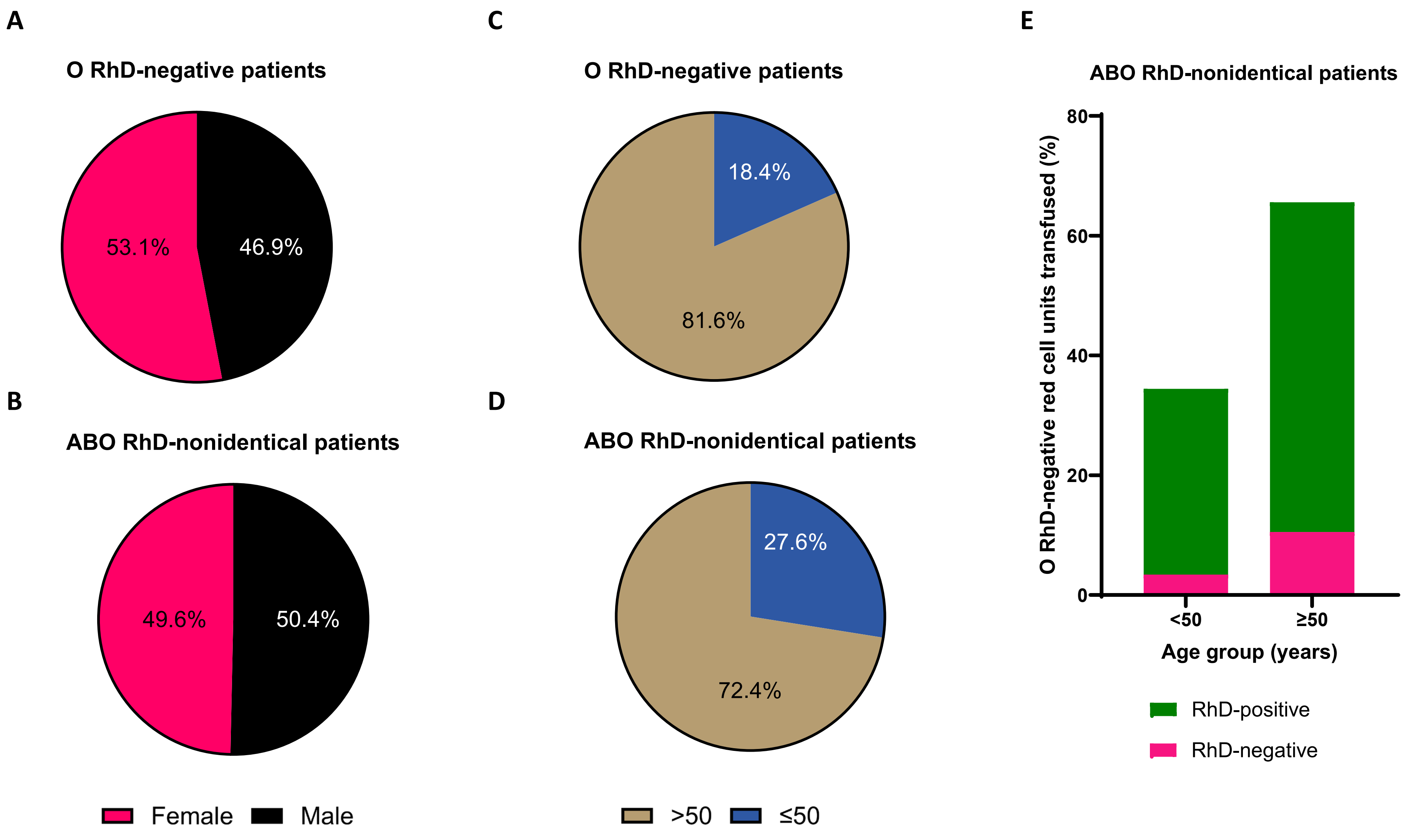
A
O RhD-negative patients
53.1%
46.9%
B
ABO RhD-nonidentical patients
49.6%
50.4%
Female
Male
C
O RhD-negative patients
18.4%
81.6%
D
ABO RhD-nonidentical patients
27.6%
72.4%
>50
≤50
E
ABO RhD-nonidentical patients
O RhD-negative red cell units transfused (%)
80
60
40
20
0
<50
≥50
Age group (years)
RhD-positive
RhD-negative

Figure S3
